# Feasibility of Using Short Message Service and In-Depth Interviews to Collect Data on Contraceptive Use Among Young, Unmarried, Sexually Active Men in Moshi, Tanzania, and Addis Ababa, Ethiopia: Mixed Methods Study With a Longitudinal Follow-Up

**DOI:** 10.2196/12657

**Published:** 2019-06-26

**Authors:** Francis Maganga Pima, Martha Oshosen, Kennedy Michael Ngowi, Bruck Messele Habte, Eusebious Maro, Belete Eshete Teffera, Godfrey Kisigo, Iraseni Ufoo Swai, Salim Semvua Msangi, Amha Ermias, Blandina T Mmbaga, Rosalijn Both, Marion Sumari-de Boer

**Affiliations:** 1 Department of Clinical Trials Kilimanjaro Clinical Research Institute Moshi United Republic of Tanzania; 2 Department of Medical Psychology University Medical Centre Amsterdam - Amsterdam Medical Centre Amsterdam Netherlands; 3 School of Pharmacy Addis Ababa University Addis Ababa Ethiopia; 4 Department of Obstetrics and Gynecology Kilimanjaro Christian Medical Centre Moshi United Republic of Tanzania; 5 SEGEL Research and Training Consulting PLC Addis Ababa Ethiopia; 6 Department of International Health Radboud University Medical Centre Nijmegen Netherlands

**Keywords:** SMS, contraceptives, sexual behavior, feasibility, young unmarried men

## Abstract

**Background:**

Data on contraceptive needs and use among young unmarried men are limited. Conventional ways of data collection may lead to limited and unreliable information on contraceptive use due to sensitivity of the topic, as many young men feel ashamed to discuss their behavior of using contraceptives. As short message service (SMS) is anonymous and a commonly used means of communication, we believe that if deployed, it will create a promising user-friendly method of data collection.

**Objective:**

The objective was to investigate the feasibility of using SMS to collect data on sexually active, young, unmarried men’s sexual behavior and contraceptive preferences, practices, and needs in Addis Ababa, Ethiopia, and Moshi, Tanzania.

**Methods:**

We enrolled men aged 18-30 years who were students (in Ethiopia and Tanzania), taxi or local bus drivers/assistants (Ethiopia and Tanzania), Kilimanjaro porters (Tanzania), or construction workers (Ethiopia). Young men were interviewed using a topic list on contraceptive use. They were followed up for 6 months by sending fortnightly SMS texts with questions about contraceptive use. If the young men indicated that they needed contraceptives during the reporting period or were not satisfied with the method they used, they were invited for a follow-up interview. At the end of the study, we conducted exit interviews telephonically using a semistructured questionnaire to explore the feasibility, acceptability, and accuracy of using SMS to validate the study findings in both countries.

**Results:**

We enrolled 71 young unmarried men—35 in Tanzania and 36 in Ethiopia. In Moshi, 1908 messages were delivered to participants and 1119 SMS responses were obtained. In Ethiopia, however, only 525 messages were sent to participants and 248 replies were received. The question on dating a girl in the past weeks was asked 438 times in Tanzania and received 252 (58%) replies, of which 148 (59%) were “YES.” In Ethiopia, this question was asked 314 times and received 64 (20%) replies, of which 52 (81%) were “YES” (*P*=.02 for difference in replies between Tanzania and Ethiopia). In Tanzania, the question on contraceptive use was sent successfully 112 times and received 108 (96%) replies, of which 105 (94%) were “YES.” In Ethiopia, the question on contraceptive use was asked 17 times and received only 2 (11%) replies. Exit interviews in Tanzania showed that SMS was accepted as a means of data collection by 22 (88%) of the 25 interviewed participants.

**Conclusions:**

Despite network and individual challenges, the SMS system was found to be feasible in Moshi, but not in Addis Ababa. We recommend more research to scale up the method in different groups and regions.

## Introduction

Data on contraceptive use is limited overall and often limited to use among girls or couples only. A study on the trends of contraceptive use from 1994 to 2014 showed that the levels of contraceptive use among women are as low as 20% in Western and Central Africa and up to 60% in Eastern and Southern Africa [1]. According to the latest Demographics and Health Surveys, in both Tanzania and Ethiopia, just over half of sexually active, unmarried women use a contraceptive method (54% in Tanzania and 55% in Ethiopia). In both countries, contraception is driven by the use of condoms, injectables, and oral contraceptive pills. In Tanzania, condoms are the most popular method used by young unmarried women (15%), while in Ethiopia, the most popular method is injectables (35%) [2,3]. The Tanzania National Family planning Research Agenda 2013-2018 reported that adolescents and young adults aged 15-24 years in Tanzania engaged in sexual activities before marriage [4], while in Ethiopia, the median age of the first sexual activity is 16-21 years [5]. Several studies have been performed in Tanzania and Ethiopia. In a study in Mwanza, Tanzania, many young men stated that they would be pleased to impregnate their partner because it demonstrates the man’s fertility [6]. In a study in an urban area in Northern Ethiopia, it was quite common among young men to frequent sex workers, and many expected their girlfriends to be virgins [7]. Nonetheless, these studies rarely touched upon the contraceptive needs and practices of these young men [8]. These studies show that there is limited information on contraceptive use and sexual behavior among young unmarried men. Collecting data through conventional ways such as face-to-face interviews or written questionnaires might not yield favorable results in this vulnerable young population.

In both Ethiopia and Tanzania, a gendered double standard allows young men much more sexual freedom than young women. Many young men in both countries claim that engaging in premarital sexual relationships prevents peers as well as women from questioning their manhood and enhances their reputation [9].

The majority of the limited existing data on contraceptive use come from demographic health surveys, other cross-sectional studies, and qualitative studies. However, due to the taboo on talking about sexual behavior and use of contraceptives, these data might be biased. Research among young, unmarried, sexually active men in countries where sexual contact before marriage is seen as a sin and is highly stigmatized needs less conventional and anonymous ways of data collection. Mobile phone usage and ownership among young men in Sub-Saharan Africa has rapidly increased in recent years [10]. Short message service (SMS) is a commonly used platform among young people for communication due to its low cost. This provides opportunities to introduce a mobile health (mHealth) app to collect or share information in order to promote knowledge on contraceptives among young men. The use of mobile phones for health is a promising way of increasing family planning knowledge and promoting a positive outlook toward contraceptive use. Furthermore, collecting data through mobile phones is possible, since access to mobile phones is relatively high. Using text messages for data collection is not expensive and unlike mass media, there is a possibility for two-way interaction. A text message with questions about contraceptive knowledge can be sent to a respondent for simple feedback, and the response can determine the follow-up question.

Several studies globally have shown the use of mobile phone surveys (MPS). A review performed by Gibson et al showed that limited information was acquired on the advantages and disadvantages of MPS, as the number of surveys using SMS is limited [11]. Another study showed that outcomes of remote data collection are too heterogenous to conclude on the feasibility of using such a tool. However, it is clear that more research is needed to investigate the feasibility of MPS [12]. Literature has shown that young people are the heaviest users of the mobile phones in their everyday life compared to other age groups [13].

To our knowledge, the SMS method has not been used to collect sensitive data about contraceptive use in our setting. As young men use mobile phones extensively, we believe SMS texts may be a promising way to generate much-needed data on the diverse and context-specific contraceptive needs, preferences, and behaviors of young men. Therefore, we conducted a qualitative study with the aim of exploring whether SMS, in combination with in-depth interviews, is a feasible method to collect data on contraceptive needs and use.

## Methods

### Study Design

This was a mixed methods study with a longitudinal follow-up. Sexually active, young, unmarried men were recruited and followed up for 6 months to collect data on sexual behavior and contraceptive use through SMS in combination with in-depth interviews. Ethical clearance for this study was obtained from the Addis Ababa Regional Health Bureau and the National Research Ethics Review Committee in Ethiopia and the local institutional review board of Kilimanjaro Christian Medical University College and the National Health Research Ethics Committee in Tanzania.

### Study Population

We recruited young, unmarried, sexually active men aged 18-29 years for our study. We involved groups of young men with diverse backgrounds regarding educational level, type of sexual partner, occupation and income, and religious affiliations. Young men recruited were students from universities (in Tanzania and Ethiopia), drivers and conductors of local transport buses (in Tanzania and Ethiopia), porters in Kilimanjaro Climbing Tourism (in Tanzania), and construction workers (in Ethiopia).

### Study Procedures

#### Recruitment

For recruitment of participants, we used different methods including posting flyers on information boards at departments in universities (for students); snowball sampling, whereby we identified one informant through our personal and professional networks who we then asked to introduce us to others in their networks; and a screening list to assess whether participants were eligible. We provided participants information about the study in Kiswahili (in Tanzania) or Amharic (in Ethiopia). After the recruiter carefully explained the nature of the study to the participant, written informed consent was obtained in Kiswahili or Amharic.

#### Informed Consent

Once a participant was recruited and signed the informed consent, we conducted an in-depth interview on his contraceptive needs and use. Participants were followed up for 6 months by sending SMS texts on a fortnightly basis to inquire about their contraceptive use in the past 2 weeks. If there was a need for contraceptives, the participants were invited for another in-depth interview that specifically focused on the instance of contraceptive need. Participants were asked about more in-depth information based on the first in-depth interview. At the end of 6-month follow-up, participants were called for exit interviews in which they were asked about the feasibility of using the SMS system.

#### Short Message Service Scheme

**Figure 1:**
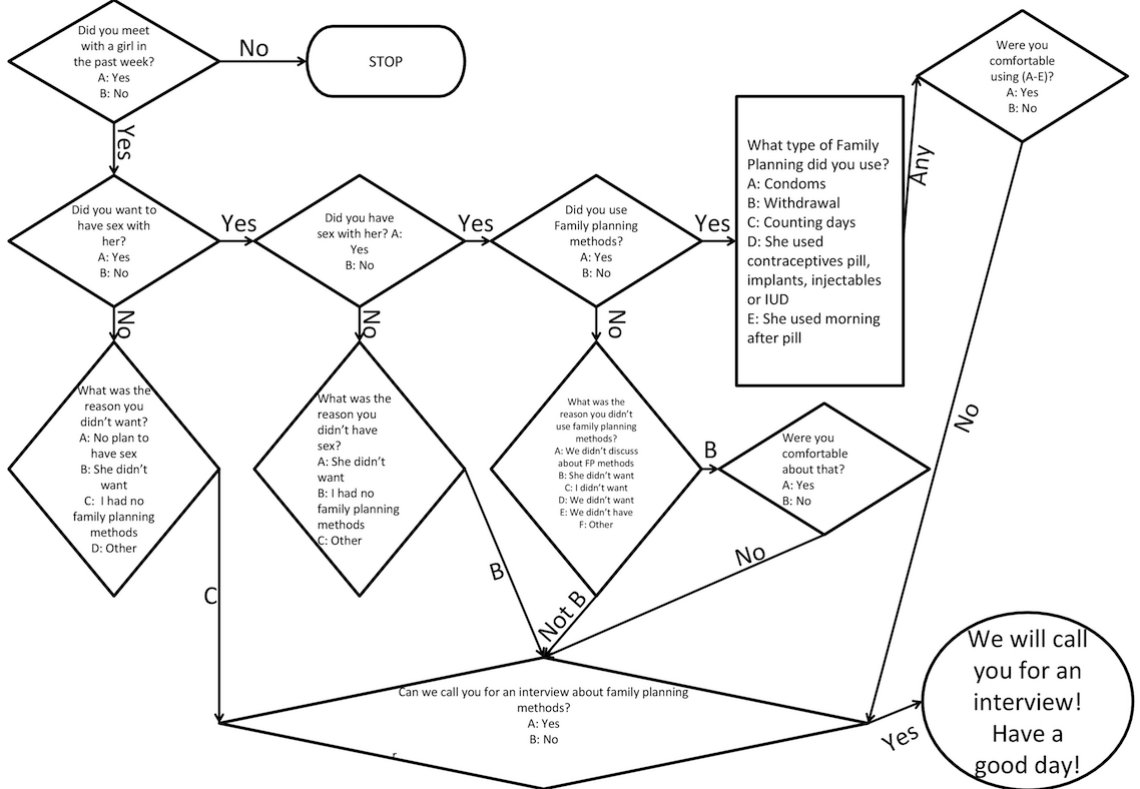
Scheme of short message service sent to participants. IUD: intrauterine device.

Participants received a fortnightly SMS message asking whether they had dated a girl in the past week. The keywords for responses were predefined, and the participants could reply via SMS. On receiving a reply, the SMS program scanned the replied message and matched it with the predefined keywords. When keywords were recognized, the program automatically sent a reply to the participant based on specified conditions and rules programmed to automatically receive another SMS. For example, if a participant replied “YES” to the question on having dated a girl, he automatically received a follow-up question on wanting to have sex with the specific girl. If he answered “NO” to the first question, he received the message, “Thank you, have a good day.” The keyword was “YES” if participant dated a girl, following which he received follow-up questions on whether he had sexual contact; whether he used contraceptives; and his reasons for not having sex, not using contraceptives, or not feeling comfortable with using contraceptives. The SMS flow is presented in Figure 1. Data from replies to the SMS texts were used to generate data about real-time sexual behavior and contraceptive needs in the past 2 weeks.

### Data Collection and Tools

#### In-Depth Interviews

After enrolment, we conducted in-depth interviews to determine contraceptive use and needs of our participants. We used a topic list on sexual behavior and contraceptive use in the past 2 weeks, number and type of partners, reasons for (non)use of contraceptives, contraceptive decision-making, use of contraceptive services, and gender dynamics. More details about these in-depth interviews will be described in the future (manuscript in preparation).

#### Technical Feasibility of Using Short Message Service

We used Telerivet software (San Francisco, California, United States) for sending SMS texts. This software generates automatic SMS texts and triggers a flow of such messages based on replies from respondents. The system generates a database containing all data and contents of the sent SMS texts, delivery reports of the texts, and replies from respondents. The Telerivet software has a standard platform that integrates with most existing mobile phone technologies and allows routing of messages to and from any number of mobile devices. It also uses a basic internet connection. With a cloud-based management system, it supports the developer to adapt an external application platform interface using other platforms for monitoring and tracking activities. The system is only accessible through authorization. Data from the system were used to calculate the technical feasibility of using SMS.

#### Follow-Up In-Depth Interviews

In order to determine the reason for nonuse of contraception for the past 2 weeks, we invited the participants to a follow-up in-depth interview, wherein we focused on the need of contraceptives for the specific sexual encounter and discomfort with the used contraceptive method, as reported by SMS texts. We first asked the participant to elaborate about the specific sexual encounter using a topic list on sexual behavior, contraceptive use in the past 2 weeks, number and type of partners, reasons for (non)use of contraceptives, contraceptive decision-making, use of contraceptive services, and gender dynamics. We also discussed other issues that arose in the enrolment in-depth interview and seemed to be of interest for our study.

#### Participants’ Perceived Feasibility

At the end of the study, we conducted exit interviews with our study participants in Moshi to inquire about their views on feasibility, acceptability, and accuracy of SMS as a data collection method. We used a semistructured questionnaire on the perceived experiences of receiving SMS, content of the SMS, difficulties in receiving the SMS, appropriateness of receiving SMS, and general views on the use of SMS to collect data on contraceptive use. We conducted exit interviews with the participants via phone calls. In Tanzania, we measured the feasibility by using a simple questionnaire on the basis of the responses obtained from those who were receiving the SMS texts on time, had experience of receiving SMS texts, were comfortable with receiving SMS texts, and thought the content was friendly. Due to the study limitations, we were unable to conduct such structured interviews in Addis Ababa. However, we asked about half of the participants how they perceived use of the SMS system.

All in-depth interviews were tape-recorded with the approval of study participants and then transcribed and translated from the local language (Swahili/Amharic) to English.

### Data Analysis

All data collected were treated confidentially and stored anonymously using code numbers in such a way that the records could not be traced back to the individuals. Code numbers (no names of participants) appeared in the database that was accessed through a secured internet account. The SMS system used was only accessible to authorized users. To avoid the risk of unwanted disclosure of sensitive information collected through SMS, we advised participants to use a password on their phones in order to avoid unwanted access. Moreover, the telecom laws of both countries maintain confidentiality of SMS texts sent through their systems.

Data from the SMS replies were analyzed using SPSS version 21.0 (IBM Corp, Armonk, NY). Descriptive analysis was conducted to explore the number of incoming and outgoing SMSs. Based on these SMS, we were able to evaluate the proportions of sent SMS texts and responses, sexual behavior, and use of contraceptives. As the focus of this study was on qualitative data, the power of the study was not adequate to make any inferential statistics. Qualitative data were analyzed through thematic framework analyses using NVivo version 11.0 (QSR International, Melbourne, Australia). After thoroughly assessing the data, codebooks were developed and refined through discussions with other study team members. Interview transcriptions were coded based on the codebook, and themes were developed in coding memos during and after completion of coding using both inductive and deductive theme extraction. These themes were linked together into larger topics that form the basis of our papers. More details will be published in the near future (manuscript in preparation). Recurrent themes will be described in more detail in our future paper on contraceptive use and knowledge, which is currently under preparation.

## Results

### Study Population

We enrolled 71 young, unmarried men—35 in Tanzania and 36 in Ethiopia. In Tanzania, we enrolled 12 porters who worked in assisting tourists climb mountain Kilimanjaro, 12 drivers of local buses (commonly known as “dala dala”), and 11 students of universities in Moshi. In Ethiopia, we enrolled 8 construction workers, 13 taxi drivers/assistants, and 15 students from different private and public higher educational institutes in Addis Ababa.

### Technological Feasibility of Using Short Message Service in Tanzania

Table 1 shows the SMS traffic for both countries. In Tanzania, a total of 3327 SMS texts were recorded, of which 1426 (43%) were among porters, 1053 (32%) were among public bus drivers, and 848 (26%) were among students. A total of 1908 (57%) of the 3327 SMS texts were sent and delivered: 802 to porters, 601 to bus drivers, and 505 to students. Moreover, we received 1119 (34%) of the 3327 SMS texts, of which 512 were from porters, 325 were from drivers, and 282 were from students. Due to no availability of network, phones being out of network, and other unknown reasons, 193 SMS texts were not delivered, 78 were queued, and 3 were ignored. Over the period of 6 months, we sent 438 SMS texts to ask whether the participant had dated a girl and we received 252 (58%) replies, of which 109 (43%) were from porters, 69 were from drivers (27%), and 74 (29%) were from students. Follow-up questions obtained a better response rate of over 96%. There were no significant differences in response rates to follow-up questions from porters, drivers, and students.

### Technological Feasibility of Using Short Message Service in Ethiopia

In Ethiopia, there was traffic of 782 SMSs, of which 525 (57%) were sent to the participants and 248 (32%) were received from the participants. Unfortunately, we have no data on whether our sent messages were received by the participants. A total of 314 SMS texts were sent to ask if the participants had dated a girl and 64 (20%) responses were obtained, of which 52 (81%) were “YES.” There was no specific information on contraceptives from the replies received from SMS texts in Ethiopia. Due to the limited feasibility of the SMS system, we decided to switch to phone data collection, and some of the participants were contacted for follow-up in this regard. There was, however, no recording of the number of phone calls made to these participants. A summary of the SMS questions sent are shown in the Table 1.

**Table 1:** Summary of the questions sent to participants via the short message service system and the responses received.

Questions sent to participants via SMS^a^		Tanzania	Ethiopia	*P* value
Total SMS traffic, n		3327	782	
Total questions delivered, n (%)		1908 (57.4)	N/A^b^	
**Question 1 delivered: Regarding whether the participant dated a girl, n (%)**		438 (22.9)	314 (40.1)	
	Responses received	252 (57.5)	64 (20.4)	<.001^c^
	Replied “YES”	148 (58.7)	52 (81.3)	.001^c^
**Question 2 delivered: Regarding whether the participant wanted to have sex, n (%)**		167 (8.8)	38 (4.9)	
	Responses received	162 (97.0)	38 (100)	.60^d^
	Replied “YES”	159 (98.1)	36 (95.0)	.20^d^
**Question 3 delivered: Regarding whether the participant had sex with a girl, n (%)**		133 (7.0)	24 (7.6)	
	Responses received	127 (95.4)	24 (100)	.60^d^
	Replied “YES”	126 (99.2)	24 (100)	>.99^d^
**Question 4 delivered: Regarding whether the participant used a contraceptive, n (%)**		112 (5.9)	17 (2.2)	
	Responses received	108 (96.4)	2 (11.8)	<.001^d^
	Replied “YES”	105 (97.2)	2 (100)	>.99^d^

^a^SMS: short message service.

^b^N/A: not applicable. There is no data on whether the messages sent were received by the participants.

^c^Chi-square test.

^d^Fisher exact test.

### Participants’ Perceived Feasibility

In Tanzania, we conducted telephonic exit interviews with 25 participants, as the others were not reachable. From the exit interviews, we found that 19 (76%) participants had a good experience with receiving SMS texts. In addition, 21 (84%) participants said they received the SMS texts on time and 22 (88%) participants had no difficulties with receiving SMS texts. A majority of the participants (n=21, 84%) found that the content of the SMS texts was good. Finally, 21 (84%) participants said it was appropriate to receive SMS texts, and 18 (72%) participants were able to respond to all the SMS texts sent to them (Multimedia Appendix 1).

In Ethiopia, based on the unstructured interviews, we found several reasons for the SMS system to be less feasible. At least five participants saw the messages but ignored them, mostly because they thought there were too many questions asked. One admitted that he responded with “NO” on purpose to the first question to avoid the next set of questions. Two men were lost to follow-up (lost their phone or did not respond to our phone calls after some time). Two taxi assistants told us they usually left their phone with others while working, while some of the construction workers had difficulties with reading Amharic and responding to the questions (although they reported being fluent in Amharic during enrolment). In addition, some participants said that the Amharic versions of the messages could not be displayed, which led to discontinuations. A couple of participants provided a phone number that was not theirs, which they only disclosed later; as such, both did not participate in the SMS system. Further, some participants responded in words instead of numbers, which were not recognized by the system. Other said they felt they received repeated messages or assumed that they were repeated messages and thus ignored them. Finally, receiving messages with errors such as jumbled order of questions or skipping of one question led to nonresponse.

### Results From In-Depth Interviews

From the recruitment and follow-up in-depth interviews, we found that our participants mostly used condoms, emergency contraception pills, and the calendar method in Addis Ababa and condoms, withdrawal, and the calendar method in Moshi. Despite existing myths on condoms, it is the most frequently used contraceptive in these regions.

Generally, the use of contraceptives depended on the knowledge, availability, cost, and community perceptions. In both countries, the use of usual hormonal contraceptives was limited, because of concerns regarding their side effects, costs, and limited knowledge on the subject. In Tanzania, we found that many young men claimed the use of hormonal contraceptives to be confined to their partners. However, many reported to have discussions with their partners prior to making a decision on the choice and use of contraception method. In most discussions, men seemed to lead the decision-making process. In Ethiopia, however, young men reported the use of emergency contraception (postpill) to be common. The majority of men reported that their partner used the postpill at least once, and it was among the most well-known methods. Textbox 1 shows a few quotations that underlie these major conclusions. Detailed results will be described in our upcoming paper.

**Example quotes from in-depth interviews.**

People, especially in the villages don’t know in details about contraceptive methods.

24-year-old college student, Tanzania

You know there are many shops in town and one can buy condoms or any other contraceptive even at night but in villages one has to walk to a distance place to search for them (condoms).

26-year-old porter, Tanzania

It is just that I fear HIV/AIDS and other STIs or else I wouldn’t use them at all.

28-year-old porter, Tanzania

Condoms are not effective, most of time they burst during the action. I and my friends had such instances.

25-year-old local town-bus driver, Tanzania

Everyone knows about it, there is no one who does not know Postpill (emergency contraceptive pill.

23-year-old student, Ethiopia

The problem with withdraw method is that you pull it out at the moment pleasure high, for me I can’t manage.

23-year-old college student, Tanzania

## Discussion

Our results show that the use of SMS for data collection seems to be feasible among young unmarried men in Moshi, Tanzania. More than half of the SMS texts received responses. However, the feasibility of using SMS in Ethiopia is questionable, as only 20% of the participants replied to the first question via SMS. Furthermore, we found that SMS and phone calls were a good entry point for collecting data on more in-depth information on sexual behaviors and contraceptive use. According to the exit interviews in Tanzania, the system was perceived to be feasible by young unmarried men.

In Tanzania, 42% of the SMS texts delivered to young men did not receive any response. One explanation could be that porters and “dala dala” drivers are often not reachable or unable to reply during work. We found a difference in response rates to the first SMS: “Dala dala” drivers responded less often than porters and students. This could be due to the fact that drivers are on duty at the time of the day when they receive the SMS texts and therefore cannot reply. There was a large difference in the response rates between Tanzania and Ethiopia. Besides the differences in cultures and the level of SMS use, participants cited several reasons for the low feasibility of the SMS system in Ethiopia. In addition, due to the technical issues at start of the study in Ethiopia, SMS texts were not always sent correctly and the participants might have been demotivated to fully participate in the study.

Two reviews have provided an overview of the advantages and disadvantages of remote data collection including SMS [11,12]. However, both studies did not conclude on whether SMS use is a good method, as the number of published studies on the use of SMS is limited. Several original studies have collected data through SMS. In another study from Tanzania that focused on the feasibility of using SMS for collecting data on contraceptive use among youth, four questions were asked on the feasibility, and the response rate ranged from 33% to 63% [13]. In Kenya, studies showed that the response rates to the text message surveys on feasibility ranged from 13.5% to 51.8%, which is lower than our results from Moshi but comparable to the results from Addis Ababa [14,15]. Similarly, in Uganda, a study reported the feasibility of using SMS in the delivery of health-related SMS texts to adolescents [16].

SMS is also used extensively in disease management and health promotion and has been shown to be effective and feasible in several studies. One study in United States showed the effectiveness of using text message for preventive sexual health promotion. Text messages in other studies have shown to be effective in helping people adhere to the clinical care management plan for chronic disease care [17,18]. In New Zealand, text messages were effective in improving adherence in asthma patients [19]. Other studies in Asia and Sub-Saharan Africa found that text message reminders were effective in improving attendance in primary care [20-22].

Our study has a few limitations. First, this is a qualitative study; the sample size was not based on power calculations, but rather on expectation of data saturation. Therefore, it is not possible to generalize the findings to other areas in Ethiopia or Tanzania, but it is likely that similar issues occur in other urban areas of Ethiopia and Tanzania as well as urban areas of other Sub-Saharan African countries.

Second, the groups that we recruited our respondents from were rather specific. Therefore, extrapolation to other groups is not recommended. However, this study generated new and valuable data that can be used for studies using quantitative research methods in larger populations. Third, due to the sensitivity of the topic, it is possible that some young men underreported their sexual activity. Finally, evidence shows that in research on sexual behaviors, use of a mixture of data collection methods results in answers that are more truthful; this is the approach we followed. We believed that by collecting data anonymously through mobile phones followed by repeated in-depth interviews and exit interviews, we would be able to build a sufficient rapport, triangulate data from each participant, and follow-up on the possible inconsistencies.

Strengths and value of the data collection method lie in the combination of data collection methods including in-depth interviews, which will serve in informing context-specific contraceptive interventions as well as the design of larger quantitative studies. Based on our findings, modifications of the systems are needed, such as the number of questions asked, shorter time of data collection, different timing of SMS texts, and a good information and communications technology structure for sending and receiving SMS texts. We recommend larger studies targeting different groups but with a shorter duration of data collection. The type of questions could also be revisited during consultation with members of the study target groups. The system that we have developed can be further modified during participatory action research, where participants can be involved in the design. Furthermore, the survey system on SMS use could be combined with educational messages on contraceptives. Other studies have also introduced use of mHealth for education and information on contraceptives [23-25]. Although in Tanzania, the mobile network coverage is high with >80%, connection to the internet is still limited. Therefore, we believe SMS, rather than a smartphone app, is an excellent way for data collection and education on contraceptive use.

In conclusion, despite network challenges and individual challenges, the SMS system for data collection was feasible in Moshi, but not in Addis Ababa; however, this system could be feasible in Addis Ababa after modifications. Therefore, in general, we believe SMS is a potential way to collect data on contraceptive use among young, unmarried men. This paper highlights the use of an innovative, client-centered way to engage young men and collect data via mobile phones, which will ultimately improve contraceptive use among young men. More evidence-based research on the use of SMS is warranted to accumulate data on the potential impact on knowledge, sexual behavior, practices, preferences, and service utilization among young men and to determine the optimal communication model for mobile phone use regarding sexual behavior and contraceptive use, which can be integrated into the national health management information system. The system should also be used to inform pharmaceutical industries on the development new contraceptive methods that meet young men’s preferences.

## Appendix

Multimedia Appendix 1 Feedback on SMS (Short Message Service) system from Tanzanian participants.

## Abbreviations

mHealth: mobile health
MPS: mobile phone surveys
N/A: not applicable
SMS: short message service
