# Supplementary material for: Feasibility of Using Short Message Service and In-Depth Interviews to Collect Data on Contraceptive Use Among Young, Unmarried, Sexually Active Men in Moshi, Tanzania, and Addis Ababa, Ethiopia: Mixed Methods Study With a Longitudinal Follow-Up
Source: JMIR Form Res. 2019 Jun 26;3(2):e12657. doi: 10.2196/12657 (PMC6617913; doi:10.2196/12657)
Supplement: Multimedia Appendix 1 [file formative_v3i2e12657_app1.pdf]

Table 2: Feedback on SMS system from Tanzanian participants

**Participants feedback from exit interviews**	**N 25(71.40%)**
**General experience with receiving SMS**	
Not good at all	0 (0.0)
Not good	1 (4.0)
Good	19 (76.0)
Very good	5 (20.0)
**Did SMS come on time?**	
Yes	21 (84.0)
No	3 (12.0)
**SMS don't come on time (n=1)**	
SMS came later as the phone was switched off	
SMS delivered late due to network problems	
**Difficulties with receiving SMS**	
Yes	3 (12.0)
No	22 (88.0)
**Difficulties**	
Some days there were no SMS received	
Delay in receiving SMS	
No airtime to reply to SMS	
**Comments about content of SMS**	

Not good at all	0 (0.0)
Not good	0 (0.0)
Good	21 (84.0)
Very good	4 (16.0)
**Any problem during receiving SMS**	
Yes	2 (8.0)
No	23 (92.0)
**Problems:**	
Issues about contraceptives were not clear	
**Is it accepted/appropriate to receive SMS?**	
Yes	21 (84)
No	4 (16.0)
**Were you able to respond to the SMS sent to you?**	
Yes	18 (72.0)
No	7 (28.0)
**Expectation on future studies on using SMS**	
There should be a sophisticated system that shows immediate reports on replies and questions asked even if network is low or unavailable	
The SMS should also focus on relationships among young men	
There should be more questions on interesting topics	
Time of sending SMS should be taken into consideration, for example it should be done after working/class hours such as late in the evening	
